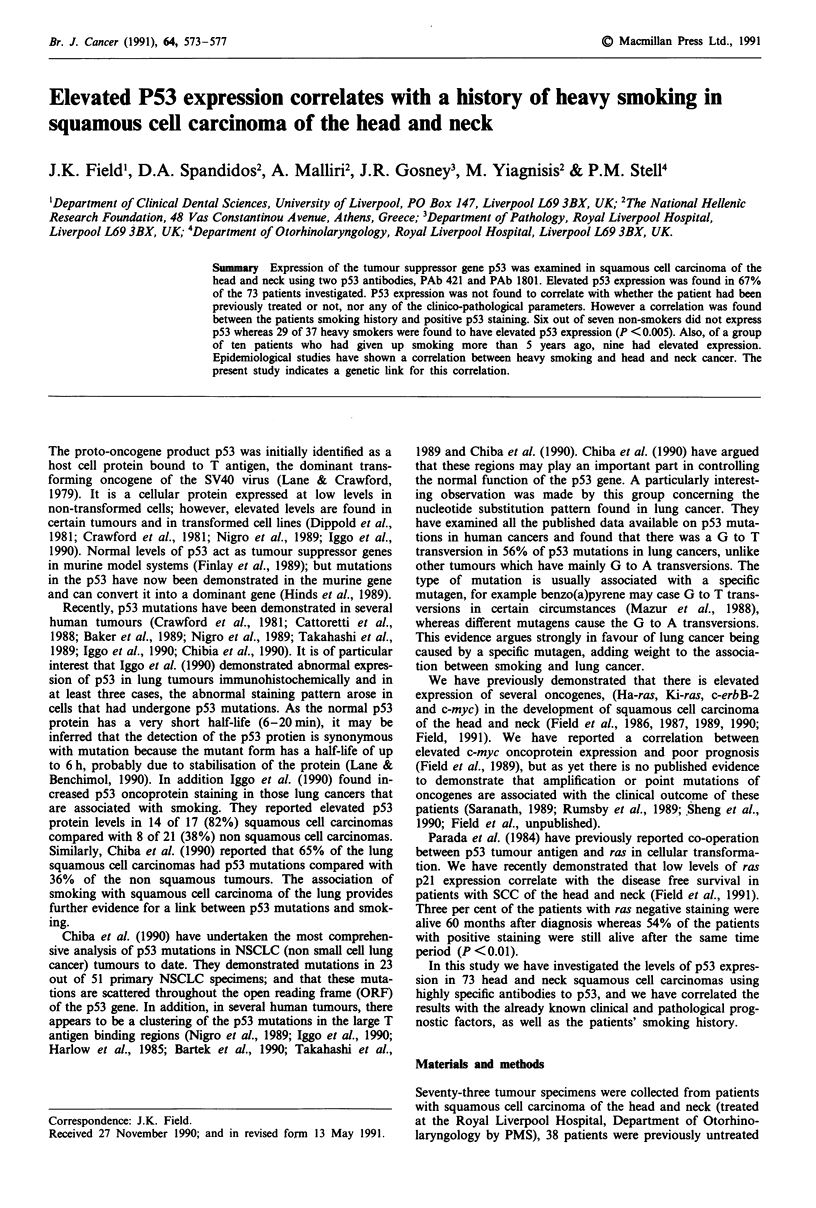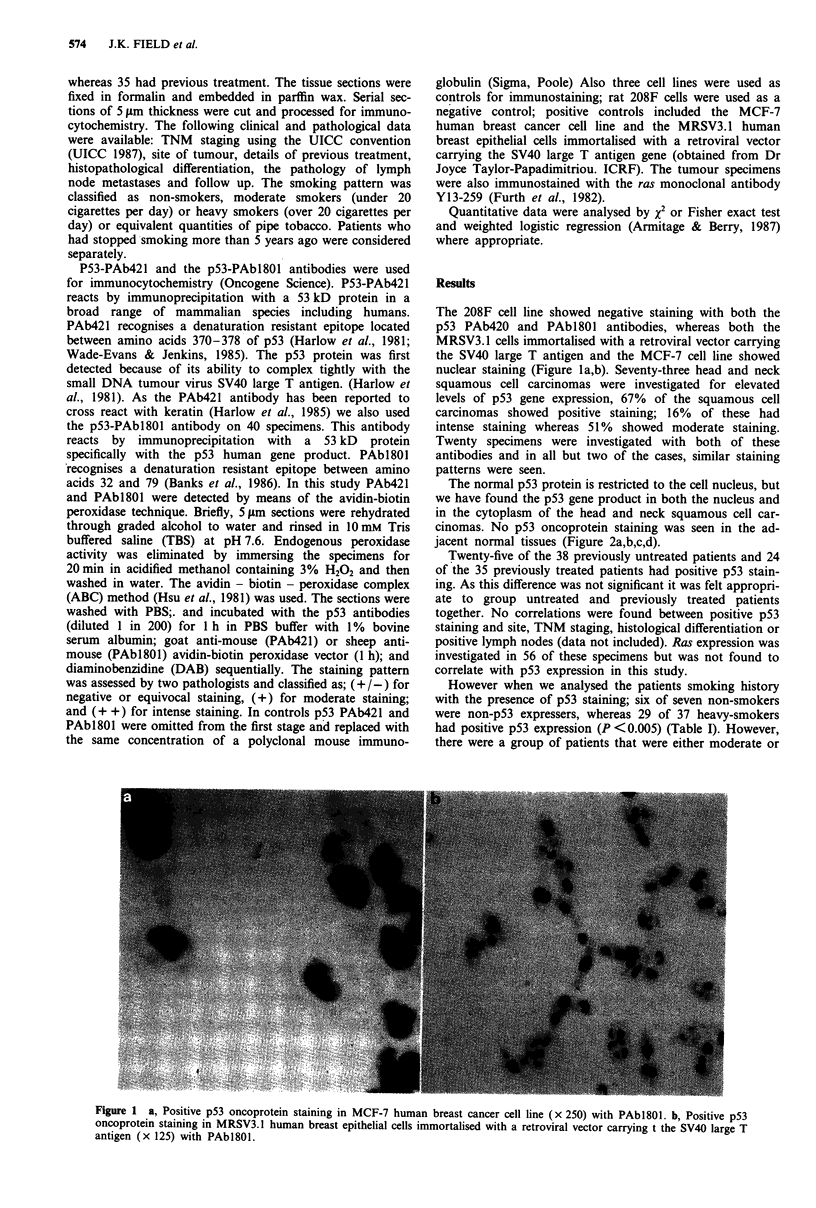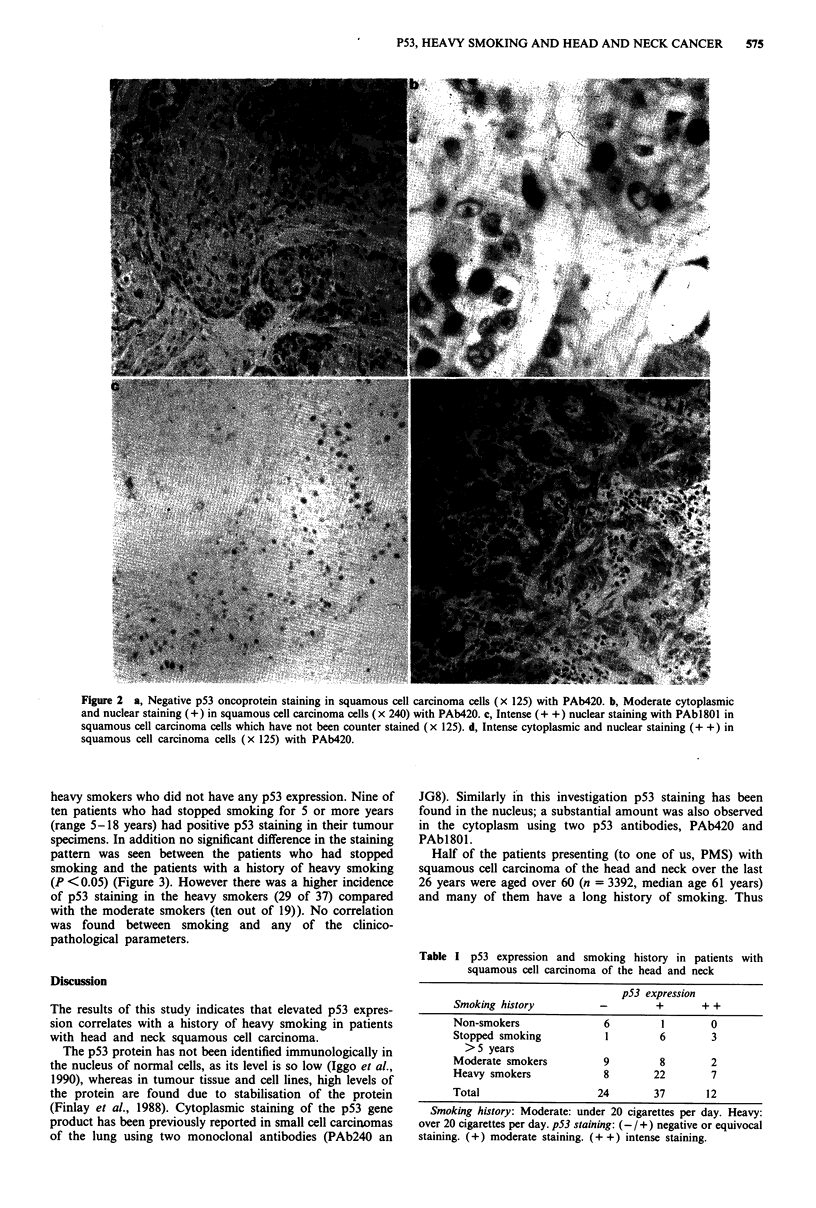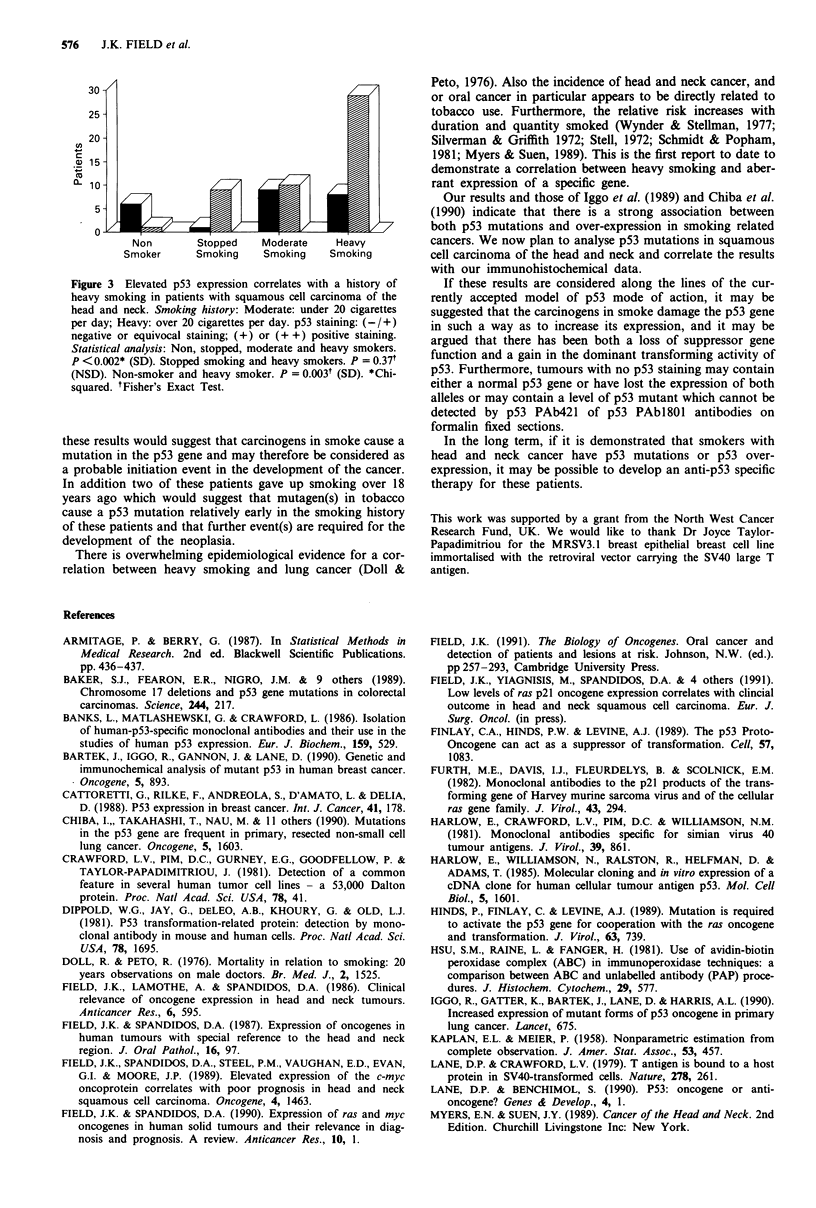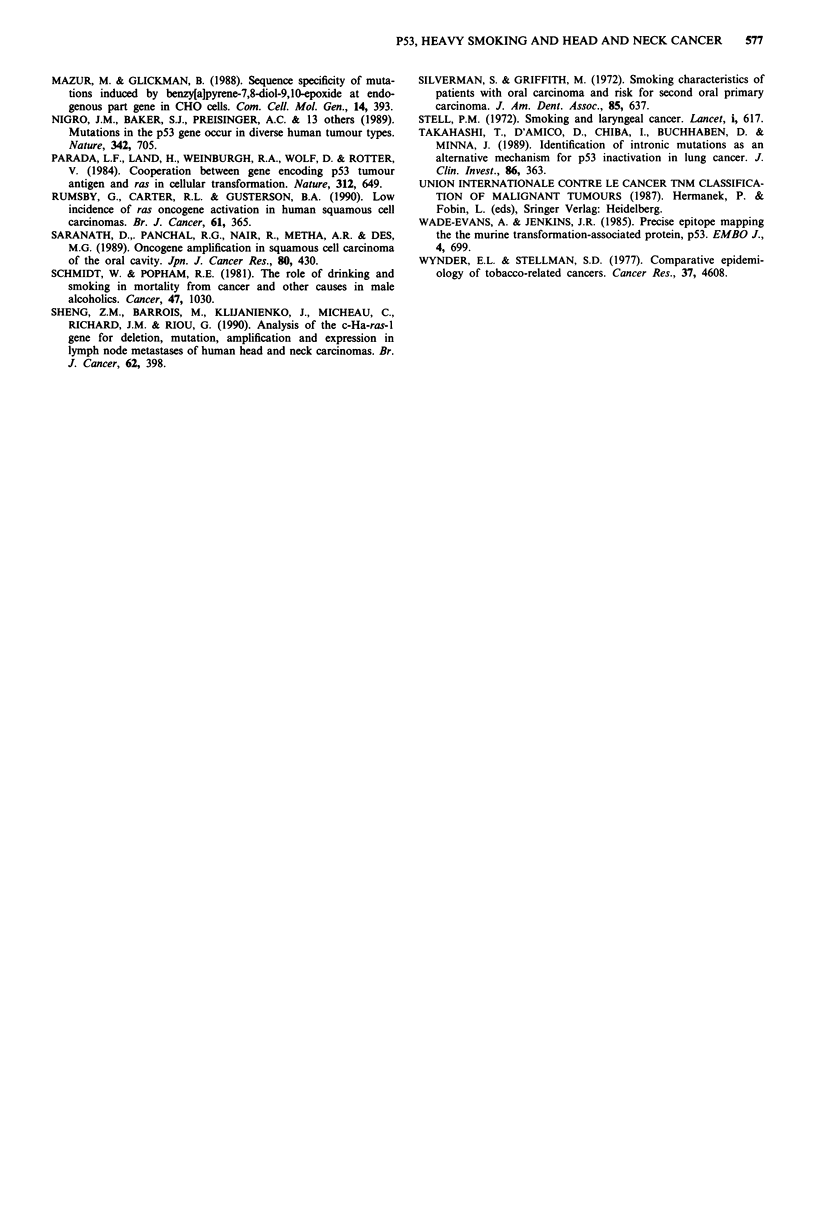# Elevated P53 expression correlates with a history of heavy smoking in squamous cell carcinoma of the head and neck.

**DOI:** 10.1038/bjc.1991.352

**Published:** 1991-09

**Authors:** J. K. Field, D. A. Spandidos, A. Malliri, J. R. Gosney, M. Yiagnisis, P. M. Stell

**Affiliations:** Department of Clinical Dental Sciences, University of Liverpool, UK.

## Abstract

**Images:**


Br. J. Cancer (1991), 64, 573-577 © Macmillan Press Ltd., 1991

### Elevated P53 expression correlates with a history of heavy smoking in squamous cell carcinoma of the head and neck

J.K. Field[1], D.A. Spandidos[2], A. Malliri[2], J.R. Gosney[3], M. Yiagnisis[2] & P.M. Stell[4]

[1]*Department of Clinical Dental Sciences, University of Liverpool, PO Box 147, Liverpool L69 3BX, UK;* [2]*The National Hellenic Research Foundation, 48 Vas Constantinou Avenue, Athens, Greece;* [3]*Department of Pathology, Royal Liverpool Hospital, Liverpool L69 3BX, UK;* [4]*Department of Otorhinolaryngology, Royal Liverpool Hospital, Liverpool L69 3BX, UK.*

**Summary** Expression of the tumour suppressor gene p53 was examined in squamous cell carcinoma of the head and neck using two p53 antibodies, PAb 421 and PAb 1801. Elevated p53 expression was found in 67% of the 73 patients investigated. P53 expression was not found to correlate with whether the patient had been previously treated or not, nor any of the clinico-pathological parameters. However a correlation was found between the patients smoking history and positive p53 staining. Six out of seven non-smokers did not express p53 whereas 29 of 37 heavy smokers were found to have elevated p53 expression ($P < 0.005$). Also, of a group of ten patients who had given up smoking more than 5 years ago, nine had elevated expression. Epidemiological studies have shown a correlation between heavy smoking and head and neck cancer. The present study indicates a genetic link for this correlation.

The proto-oncogene product p53 was initially identified as a host cell protein bound to T antigen, the dominant transforming oncogene of the SV40 virus (Lane & Crawford, 1979). It is a cellular protein expressed at low levels in non-transformed cells; however, elevated levels are found in certain tumours and in transformed cell lines (Dippold *et al.*, 1981; Crawford *et al.*, 1981; Nigro *et al.*, 1989; Iggo *et al.*, 1990). Normal levels of p53 act as tumour suppressor genes in murine model systems (Finlay *et al.*, 1989); but mutations in the p53 have now been demonstrated in the murine gene and can convert it into a dominant gene (Hinds *et al.*, 1989).

Recently, p53 mutations have been demonstrated in several human tumours (Crawford *et al.*, 1981; Cattoretti *et al.*, 1988; Baker *et al.*, 1989; Nigro *et al.*, 1989; Takahashi *et al.*, 1989; Iggo *et al.*, 1990; Chibia *et al.*, 1990). It is of particular interest that Iggo *et al.* (1990) demonstrated abnormal expression of p53 in lung tumours immunohistochemically and in at least three cases, the abnormal staining pattern arose in cells that had undergone p53 mutations. As the normal p53 protein has a very short half-life (6-20 min), it may be inferred that the detection of the p53 protien is synonymous with mutation because the mutant form has a half-life of up to 6 h, probably due to stabilisation of the protein (Lane & Benchimol, 1990). In addition Iggo *et al.* (1990) found increased p53 oncoprotein staining in those lung cancers that are associated with smoking. They reported elevated p53 protein levels in 14 of 17 (82%) squamous cell carcinomas compared with 8 of 21 (38%) non squamous cell carcinomas. Similarly, Chiba *et al.* (1990) reported that 65% of the lung squamous cell carcinomas had p53 mutations compared with 36% of the non squamous tumours. The association of smoking with squamous cell carcinoma of the lung provides further evidence for a link between p53 mutations and smoking.

Chiba *et al.* (1990) have undertaken the most comprehensive analysis of p53 mutations in NSCLC (non small cell lung cancer) tumours to date. They demonstrated mutations in 23 out of 51 primary NSCLC specimens; and that these mutations are scattered throughout the open reading frame (ORF) of the p53 gene. In addition, in several human tumours, there appears to be a clustering of the p53 mutations in the large T antigen binding regions (Nigro *et al.*, 1989; Iggo *et al.*, 1990; Harlow *et al.*, 1985; Bartek *et al.*, 1990; Takahashi *et al.*, 1989 and Chiba *et al.* (1990). Chiba *et al.* (1990) have argued that these regions may play an important part in controlling the normal function of the p53 gene. A particularly interesting observation was made by this group concerning the nucleotide substitution pattern found in lung cancer. They have examined all the published data available on p53 mutations in human cancers and found that there was a G to T transversion in 56% of p53 mutations in lung cancers, unlike other tumours which have mainly G to A transversions. The type of mutation is usually associated with a specific mutagen, for example benzo(a)pyrene may case G to T transversions in certain circumstances (Mazur *et al.*, 1988), whereas different mutagens cause the G to A transversions. This evidence argues strongly in favour of lung cancer being caused by a specific mutagen, adding weight to the association between smoking and lung cancer.

We have previously demonstrated that there is elevated expression of several oncogenes, (Ha-*ras*, Ki-*ras*, c-*erb*B-2 and c-*myc*) in the development of squamous cell carcinoma of the head and neck (Field *et al.*, 1986, 1987, 1989, 1990; Field, 1991). We have reported a correlation between elevated c-*myc* oncoprotein expression and poor prognosis (Field *et al.*, 1989), but as yet there is no published evidence to demonstrate that amplification or point mutations of oncogenes are associated with the clinical outcome of these patients (Saranath, 1989; Rumsby *et al.*, 1989; Sheng *et al.*, 1990; Field *et al.*, unpublished).

Parada *et al.* (1984) have previously reported co-operation between p53 tumour antigen and *ras* in cellular transformation. We have recently demonstrated that low levels of *ras* p21 expression correlate with the disease free survival in patients with SCC of the head and neck (Field *et al.*, 1991). Three per cent of the patients with *ras* negative staining were alive 60 months after diagnosis whereas 54% of the patients with positive staining were still alive after the same time period ($P < 0.01$).

In this study we have investigated the levels of p53 expression in 73 head and neck squamous cell carcinomas using highly specific antibodies to p53, and we have correlated the results with the already known clinical and pathological prognostic factors, as well as the patients' smoking history.

### Materials and methods

Seventy-three tumour specimens were collected from patients with squamous cell carcinoma of the head and neck (treated at the Royal Liverpool Hospital, Department of Otorhinolaryngology by PMS), 38 patients were previously untreated

Correspondence: J.K. Field.
Received 27 November 1990; and in revised form 13 May 1991.

whereas 35 had previous treatment. The tissue sections were fixed in formalin and embedded in parffin wax. Serial sections of 5 μm thickness were cut and processed for immunocytochemistry. The following clinical and pathological data were available: TNM staging using the UICC convention (UICC 1987), site of tumour, details of previous treatment, histopathological differentiation, the pathology of lymph node metastases and follow up. The smoking pattern was classified as non-smokers, moderate smokers (under 20 cigarettes per day) or heavy smokers (over 20 cigarettes per day) or equivalent quantities of pipe tobacco. Patients who had stopped smoking more than 5 years ago were considered separately.

P53-PAb421 and the p53-PAb1801 antibodies were used for immunocytochemistry (Oncogene Science). P53-PAb421 reacts by immunoprecipitation with a 53 kD protein in a broad range of mammalian species including humans. PAb421 recognises a denaturation resistant epitope located between amino acids 370–378 of p53 (Harlow *et al.*, 1981; Wade-Evans & Jenkins, 1985). The p53 protein was first detected because of its ability to complex tightly with the small DNA tumour virus SV40 large T antigen. (Harlow *et al.*, 1981). As the PAb421 antibody has been reported to cross react with keratin (Harlow *et al.*, 1985) we also used the p53-PAb1801 antibody on 40 specimens. This antibody reacts by immunoprecipitation with a 53 kD protein specifically with the p53 human gene product. PAb1801 recognises a denaturation resistant epitope between amino acids 32 and 79 (Banks *et al.*, 1986). In this study PAb421 and PAb1801 were detected by means of the avidin-biotin peroxidase technique. Briefly, 5 μm sections were rehydrated through graded alcohol to water and rinsed in 10 mM Tris buffered saline (TBS) at pH 7.6. Endogenous peroxidase activity was eliminated by immersing the specimens for 20 min in acidified methanol containing 3% $H_2O_2$ and then washed in water. The avidin – biotin – peroxidase complex (ABC) method (Hsu *et al.*, 1981) was used. The sections were washed with PBS;. and incubated with the p53 antibodies (diluted 1 in 200) for 1 h in PBS buffer with 1% bovine serum albumin; goat anti-mouse (PAb421) or sheep anti-mouse (PAb1801) avidin-biotin peroxidase vector (1 h); and diaminobenzidine (DAB) sequentially. The staining pattern was assessed by two pathologists and classified as; (+/−) for negative or equivocal staining, (+) for moderate staining; and (+ +) for intense staining. In controls p53 PAb421 and PAb1801 were omitted from the first stage and replaced with the same concentration of a polyclonal mouse immunoglobulin (Sigma, Poole) Also three cell lines were used as controls for immunostaining; rat 208F cells were used as a negative control; positive controls included the MCF-7 human breast cancer cell line and the MRSV3.1 human breast epithelial cells immortalised with a retroviral vector carrying the SV40 large T antigen gene (obtained from Dr Joyce Taylor-Papadimitriou. ICRF). The tumour specimens were also immunostained with the *ras* monoclonal antibody Y13-259 (Furth *et al.*, 1982).

Quantitative data were analysed by $\chi^2$ or Fisher exact test and weighted logistic regression (Armitage & Berry, 1987) where appropriate.

### Results

The 208F cell line showed negative staining with both the p53 PAb420 and PAb1801 antibodies, whereas both the MRSV3.1 cells immortalised with a retroviral vector carrying the SV40 large T antigen and the MCF-7 cell line showed nuclear staining (Figure 1a,b). Seventy-three head and neck squamous cell carcinomas were investigated for elevated levels of p53 gene expression, 67% of the squamous cell carcinomas showed positive staining; 16% of these had intense staining whereas 51% showed moderate staining. Twenty specimens were investigated with both of these antibodies and in all but two of the cases, similar staining patterns were seen.

The normal p53 protein is restricted to the cell nucleus, but we have found the p53 gene product in both the nucleus and in the cytoplasm of the head and neck squamous cell carcinomas. No p53 oncoprotein staining was seen in the adjacent normal tissues (Figure 2a,b,c,d).

Twenty-five of the 38 previously untreated patients and 24 of the 35 previously treated patients had positive p53 staining. As this difference was not significant it was felt appropriate to group untreated and previously treated patients together. No correlations were found between positive p53 staining and site, TNM staging, histological differentiation or positive lymph nodes (data not included). *Ras* expression was investigated in 56 of these specimens but was not found to correlate with p53 expression in this study.

However when we analysed the patients smoking history with the presence of p53 staining; six of seven non-smokers were non-p53 expressers, whereas 29 of 37 heavy-smokers had positive p53 expression ($P < 0.005$) (Table I). However, there were a group of patients that were either moderate or

**Figure 1** **a**, Positive p53 oncoprotein staining in MCF-7 human breast cancer cell line (× 250) with PAb1801. **b**, Positive p53 oncoprotein staining in MRSV3.1 human breast epithelial cells immortalised with a retroviral vector carrying t the SV40 large T antigen (× 125) with PAb1801.

**Figure 2** **a**, Negative p53 oncoprotein staining in squamous cell carcinoma cells (× 125) with PAb420. **b**, Moderate cytoplasmic and nuclear staining (+) in squamous cell carcinoma cells (× 240) with PAb420. **c**, Intense (+ +) nuclear staining with PAb1801 in squamous cell carcinoma cells which have not been counter stained (× 125). **d**, Intense cytoplasmic and nuclear staining (+ +) in squamous cell carcinoma cells (× 125) with PAb420.

heavy smokers who did not have any p53 expression. Nine of ten patients who had stopped smoking for 5 or more years (range 5–18 years) had positive p53 staining in their tumour specimens. In addition no significant difference in the staining pattern was seen between the patients who had stopped smoking and the patients with a history of heavy smoking ($P < 0.05$) (Figure 3). However there was a higher incidence of p53 staining in the heavy smokers (29 of 37) compared with the moderate smokers (ten out of 19)). No correlation was found between smoking and any of the clinico-pathological parameters.

### Discussion

The results of this study indicates that elevated p53 expression correlates with a history of heavy smoking in patients with head and neck squamous cell carcinoma.

The p53 protein has not been identified immunologically in the nucleus of normal cells, as its level is so low (Iggo *et al.*, 1990), whereas in tumour tissue and cell lines, high levels of the protein are found due to stabilisation of the protein (Finlay *et al.*, 1988). Cytoplasmic staining of the p53 gene product has been previously reported in small cell carcinomas of the lung using two monoclonal antibodies (PAb240 an JG8). Similarly in this investigation p53 staining has been found in the nucleus; a substantial amount was also observed in the cytoplasm using two p53 antibodies, PAb420 and PAb1801.

Half of the patients presenting (to one of us, PMS) with squamous cell carcinoma of the head and neck over the last 26 years were aged over 60 ($n$ = 3392, median age 61 years) and many of them have a long history of smoking. Thus

**Table I** p53 expression and smoking history in patients with squamous cell carcinoma of the head and neck

| *Smoking history* | *p53 expression* – | + | + + |
|---|---|---|---|
| Non-smokers | 6 | 1 | 0 |
| Stopped smoking >5 years | 1 | 6 | 3 |
| Moderate smokers | 9 | 8 | 2 |
| Heavy smokers | 8 | 22 | 7 |
| Total | 24 | 37 | 12 |

*Smoking history*: Moderate: under 20 cigarettes per day. Heavy: over 20 cigarettes per day. *p53 staining*: (–/+) negative or equivocal staining. (+) moderate staining. (+ +) intense staining.

**Figure 3** Elevated p53 expression correlates with a history of heavy smoking in patients with squamous cell carcinoma of the head and neck. *Smoking history*: Moderate: under 20 cigarettes per day; Heavy: over 20 cigarettes per day. p53 staining: (−/+) negative or equivocal staining; (+) or (++) positive staining. *Statistical analysis*: Non, stopped, moderate and heavy smokers. $P < 0.002^{*}$ (SD). Stopped smoking and heavy smokers. $P = 0.37^{\dagger}$ (NSD). Non-smoker and heavy smoker. $P = 0.003^{\dagger}$ (SD). *Chi-squared. †Fisher's Exact Test.

these results would suggest that carcinogens in smoke cause a mutation in the p53 gene and may therefore be considered as a probable initiation event in the development of the cancer. In addition two of these patients gave up smoking over 18 years ago which would suggest that mutagen(s) in tobacco cause a p53 mutation relatively early in the smoking history of these patients and that further event(s) are required for the development of the neoplasia.

There is overwhelming epidemiological evidence for a correlation between heavy smoking and lung cancer (Doll & Peto, 1976). Also the incidence of head and neck cancer, and or oral cancer in particular appears to be directly related to tobacco use. Furthermore, the relative risk increases with duration and quantity smoked (Wynder & Stellman, 1977; Silverman & Griffith 1972; Stell, 1972; Schmidt & Popham, 1981; Myers & Suen, 1989). This is the first report to date to demonstrate a correlation between heavy smoking and aberrant expression of a specific gene.

Our results and those of Iggo *et al.* (1989) and Chiba *et al.* (1990) indicate that there is a strong association between both p53 mutations and over-expression in smoking related cancers. We now plan to analyse p53 mutations in squamous cell carcinoma of the head and neck and correlate the results with our immunohistochemical data.

If these results are considered along the lines of the currently accepted model of p53 mode of action, it may be suggested that the carcinogens in smoke damage the p53 gene in such a way as to increase its expression, and it may be argued that there has been both a loss of suppressor gene function and a gain in the dominant transforming activity of p53. Furthermore, tumours with no p53 staining may contain either a normal p53 gene or have lost the expression of both alleles or may contain a level of p53 mutant which cannot be detected by p53 PAb421 of p53 PAb1801 antibodies on formalin fixed sections.

In the long term, if it is demonstrated that smokers with head and neck cancer have p53 mutations or p53 over-expression, it may be possible to develop an anti-p53 specific therapy for these patients.

This work was supported by a grant from the North West Cancer Research Fund, UK. We would like to thank Dr Joyce Taylor-Papadimitriou for the MRSV3.1 breast epithelial breast cell line immortalised with the retroviral vector carrying the SV40 large T antigen.

### References

ARMITAGE, P. & BERRY, G. (1987). In *Statistical Methods in Medical Research*. 2nd ed. Blackwell Scientific Publications. pp. 436–437.

BAKER, S.J., FEARON, E.R., NIGRO, J.M. & 9 others (1989). Chromosome 17 deletions and p53 gene mutations in colorectal carcinomas. *Science*, **244**, 217.

BANKS, L., MATLASHEWSKI, G. & CRAWFORD, L. (1986). Isolation of human-p53-specific monoclonal antibodies and their use in the studies of human p53 expression. *Eur. J. Biochem.*, **159**, 529.

BARTEK, J., IGGO, R., GANNON, J. & LANE, D. (1990). Genetic and immunochemical analysis of mutant p53 in human breast cancer. *Oncogene*, **5**, 893.

CATTORETTI, G., RILKE, F., ANDREOLA, S., D'AMATO, L. & DELIA, D. (1988). P53 expression in breast cancer. *Int. J. Cancer*, **41**, 178.

CHIBA, I.,, TAKAHASHI, T., NAU, M. & 11 others (1990). Mutations in the p53 gene are frequent in primary, resected non-small cell lung cancer. *Oncogene*, **5**, 1603.

CRAWFORD, L.V., PIM, D.C., GURNEY, E.G., GOODFELLOW, P. & TAYLOR-PAPADIMITRIOU, J. (1981). Detection of a common feature in several human tumor cell lines – a 53,000 Dalton protein. *Proc. Natl Acad. Sci. USA*, **78**, 41.

DIPPOLD, W.G., JAY, G., DELEO, A.B., KHOURY, G. & OLD, L.J. (1981). P53 transformation-related protein: detection by monoclonal antibody in mouse and human cells. *Proc. Natl Acad. Sci. USA*, **78**, 1695.

DOLL, R. & PETO, R. (1976). Mortality in relation to smoking: 20 years observations on male doctors. *Br. Med. J.*, **2**, 1525.

FIELD, J.K., LAMOTHE, A. & SPANDIDOS, D.A. (1986). Clinical relevance of oncogene expression in head and neck tumours. *Anticancer Res.*, **6**, 595.

FIELD, J.K. & SPANDIDOS, D.A. (1987). Expression of oncogenes in human tumours with special reference to the head and neck region. *J. Oral Pathol.*, **16**, 97.

FIELD, J.K., SPANDIDOS, D.A., STEEL, P.M., VAUGHAN, E.D., EVAN, G.I. & MOORE, J.P. (1989). Elevated expression of the *c-myc* oncoprotein correlates with poor prognosis in head and neck squamous cell carcinoma. *Oncogene*, **4**, 1463.

FIELD, J.K. & SPANDIDOS, D.A. (1990). Expression of *ras* and *myc* oncogenes in human solid tumours and their relevance in diagnosis and prognosis. A review. *Anticancer Res.*, **10**, 1.

FIELD, J.K. (1991). *The Biology of Oncogenes*. Oral cancer and detection of patients and lesions at risk. Johnson, N.W. (ed.). pp 257–293, Cambridge University Press.

FIELD, J.K., YIAGNISIS, M., SPANDIDOS, D.A. & 4 others (1991). Low levels of *ras* p21 oncogene expression correlates with clincial outcome in head and neck squamous cell carcinoma. *Eur. J. Surg. Oncol.* (in press).

FINLAY, C.A., HINDS, P.W. & LEVINE, A.J. (1989). The p53 Proto-Oncogene can act as a suppressor of transformation. *Cell*, **57**, 1083.

FURTH, M.E., DAVIS, I.J., FLEURDELYS, B. & SCOLNICK, E.M. (1982). Monoclonal antibodies to the p21 products of the transforming gene of Harvey murine sarcoma virus and of the cellular *ras* gene family. *J. Virol.*, **43**, 294.

HARLOW, E., CRAWFORD, L.V., PIM, D.C. & WILLIAMSON, N.M. (1981). Monoclonal antibodies specific for simian virus 40 tumour antigens. *J. Virol.*, **39**, 861.

HARLOW, E., WILLIAMSON, N., RALSTON, R., HELFMAN, D. & ADAMS, T. (1985). Molecular cloning and *in vitro* expression of a cDNA clone for human cellular tumour antigen p53. *Mol. Cell Biol.*, **5**, 1601.

HINDS, P., FINLAY, C. & LEVINE, A.J. (1989). Mutation is required to activate the p53 gene for cooperation with the *ras* oncogene and transformation. *J. Virol.*, **63**, 739.

HSU, S.M., RAINE, L. & FANGER, H. (1981). Use of avidin-biotin peroxidase complex (ABC) in immunoperoxidase techniques: a comparison between ABC and unlabelled antibody (PAP) procedures. *J. Histochem. Cytochem.*, **29**, 577.

IGGO, R., GATTER, K., BARTEK, J., LANE, D. & HARRIS, A.L. (1990). Increased expression of mutant forms of p53 oncogene in primary lung cancer. *Lancet*, 675.

KAPLAN, E.L. & MEIER, P. (1958). Nonparametric estimation from complete observation. *J. Amer. Stat. Assoc.*, **53**, 457.

LANE, D.P. & CRAWFORD, L.V. (1979). T antigen is bound to a host protein in SV40-transformed cells. *Nature*, **278**, 261.

LANE, D.P. & BENCHIMOL, S. (1990). P53: oncogene or anti-oncogene? *Genes & Develop.*, **4**, 1.

MYERS, E.N. & SUEN, J.Y. (1989). *Cancer of the Head and Neck*. 2nd Edition. Churchill Livingstone Inc: New York.

MAZUR, M. & GLICKMAN, B. (1988). Sequence specificity of mutations induced by benzy[a]pyrene-7,8-diol-9,10-epoxide at endogenous part gene in CHO cells. *Com. Cell. Mol. Gen.*, **14**, 393.

NIGRO, J.M., BAKER, S.J., PREISINGER, A.C. & 13 others (1989). Mutations in the p53 gene occur in diverse human tumour types. *Nature*, **342**, 705.

PARADA, L.F., LAND, H., WEINBURGH, R.A., WOLF, D. & ROTTER, V. (1984). Cooperation between gene encoding p53 tumour antigen and *ras* in cellular transformation. *Nature*, **312**, 649.

RUMSBY, G., CARTER, R.L. & GUSTERSON, B.A. (1990). Low incidence of *ras* oncogene activation in human squamous cell carcinomas. *Br. J. Cancer*, **61**, 365.

SARANATH, D.,. PANCHAL, R.G., NAIR, R., METHA, A.R. & DES, M.G. (1989). Oncogene amplification in squamous cell carcinoma of the oral cavity. *Jpn. J. Cancer Res.*, **80**, 430.

SCHMIDT, W. & POPHAM, R.E. (1981). The role of drinking and smoking in mortality from cancer and other causes in male alcoholics. *Cancer*, **47**, 1030.

SHENG, Z.M., BARROIS, M., KLIJANIENKO, J., MICHEAU, C., RICHARD, J.M. & RIOU, G. (1990). Analysis of the c-Ha-*ras*-1 gene for deletion, mutation, amplification and expression in lymph node metastases of human head and neck carcinomas. *Br. J. Cancer*, **62**, 398.

SILVERMAN, S. & GRIFFITH, M. (1972). Smoking characteristics of patients with oral carcinoma and risk for second oral primary carcinoma. *J. Am. Dent. Assoc.*, **85**, 637.

STELL, P.M. (1972). Smoking and laryngeal cancer. *Lancet*, **i**, 617.

TAKAHASHI, T., D'AMICO, D., CHIBA, I., BUCHHABEN, D. & MINNA, J. (1989). Identification of intronic mutations as an alternative mechanism for p53 inactivation in lung cancer. *J. Clin. Invest.*, **86**, 363.

UNION INTERNATIONALE CONTRE LE CANCER TNM CLASSIFICATION OF MALIGNANT TUMOURS (1987). Hermanek, P. & Fobin, L. (eds), Sringer Verlag: Heidelberg.

WADE-EVANS, A. & JENKINS, J.R. (1985). Precise epitope mapping the the murine transformation-associated protein, p53. *EMBO J.*, **4**, 699.

WYNDER, E.L. & STELLMAN, S.D. (1977). Comparative epidemiology of tobacco-related cancers. *Cancer Res.*, **37**, 4608.